# Magnetic Nanocarriers with ICPTES- and GPTMS-Functionalized Quaternary Chitosan for pH-Responsive Doxorubicin Release

**DOI:** 10.3390/biom16010137

**Published:** 2026-01-13

**Authors:** Sofia F. Soares, Ana L. M. Machado, Beatriz S. Cardoso, Diogo Marinheiro, Nelson Andrade, Fátima Martel, Ana L. Daniel-da-Silva

**Affiliations:** 1 CICECO-Aveiro Institute of Materials, Department of Chemistry, University of Aveiro, 3810-193 Aveiro, Portugal; analuisamachado@ua.pt (A.L.M.M.); b.cardoso@ua.pt (B.S.C.); diogomarinheiro@ua.pt (D.M.); 2 Unit of Biochemistry, Department of Biomedicine, Faculty of Medicine of Porto, University of Porto, 4200-319 Porto, Portugal; nandrade@med.up.pt (N.A.); fmartel@med.up.pt (F.M.); 3 REQUIMTE/LAQV, Department of Chemical Sciences, Faculty of Pharmacy, University of Porto, 4050-313 Porto, Portugal; 4 Instituto de Investigação e Inovação em Saúde (i3S), University of Porto, 4200-465 Porto, Portugal

**Keywords:** magnetic nanocarriers, quaternary chitosan, doxorubicin release, pH-responsive drug delivery, surface functionalization

## Abstract

Smart nanocarriers are being increasingly explored to improve the performance selectivity of cancer chemotherapy. Here, two pH-responsive magnetic nanocarriers were developed using quaternary chitosan (HTCC) functionalized with 3-(triethoxysilyl)propyl isocyanate- ICPTES (MNP-HTCC1) or 3-(glycidyloxypropyl)trimethoxysilane-GPTMS (MNP-HTCC2) to form hybrid silica shells on Fe_3_O_4_ cores. The resulting core–shell nanoparticles (14.5 and 12.5 nm) displayed highly positive zeta potentials (+45.4 to +27.1 mV, pH 4.2–9.5), confirming successful HTCC incorporation and strong colloidal stability. Both nanocarriers achieved high doxorubicin (DOX) loading at pH 9.5, reaching 90% efficiency and a capacity of 154 µg DOX per mg. DOX release was pH-dependent, with faster release under acidic conditions relevant to tumor and endo-lysosomal environments. At pH 4.2, MNP-HTCC1 released 90% of DOX over 72 h, while MNP-HTCC2 released 79%. Release at pH 5.0 was intermediate (67–72%), and moderate at physiological pH (43–55%). All formulations showed an initial burst followed by sustained release. Kinetic modelling (Weibull) indicated a diffusion-controlled mechanism consistent with Fickian transport through the HTCC–silica matrix. Cytotoxicity assays using MCF-7 breast cancer cells revealed greater cytotoxicity for DOX-loaded nanocarriers compared with free DOX, with MNP-HTCC1 showing the strongest effect. Overall, these HTCC-based magnetic nanocarriers offer efficient loading, controlled pH-triggered DOX release, and enhanced therapeutic performance.

## 1. Introduction

Cancer remains one of the leading causes of mortality worldwide. Among various treatment approaches for solid tumors, surgery is typically the primary intervention, often followed by chemotherapy and radiotherapy to prevent recurrence and metastasis [1]. However, the therapeutic efficacy of conventional chemotherapeutic agents, such as doxorubicin (DOX), is often hampered by severe systemic toxicity, poor selectivity, and multidrug resistance [2,3,4]. These off-target effects not only damage malignant cells but also harm healthy tissues, leading to serious complications including cardiotoxicity, cognitive impairment, and hepatotoxicity [5], which impose strict dose restrictions and ultimately compromise treatment outcomes. These limitations highlight the need for advanced drug delivery systems capable of enhancing DOX selectivity while minimizing systemic toxicity [6].

Nanocarrier-assisted drug delivery systems have shown strong potential to enhance tumor targeting, improve pharmacokinetics, and minimize off-target effects [7,8,9]. Magnetic nanoparticles (MNPs), in particular magnetite (Fe_3_O_4_), offer additional advantages, including superparamagnetism, biocompatibility, facile surface modification, and the possibility of magnetic guidance or hyperthermia [10,11,12,13]. The unique combination of magnetic properties and chemical versatility makes Fe_3_O_4_ nanoparticles especially valuable in biomedical applications. Their superparamagnetic nature allows for remote control and targeting using external magnetic fields, while their large surface area enables efficient drug loading, surface functionalization, and conjugation with targeting ligands [14]. Consequently, Fe_3_O_4_ nanoparticles have been widely explored for applications such as targeted drug delivery, magnetic resonance imaging (MRI) contrast enhancement, magnetic hyperthermia for tumor ablation, and biosensing, demonstrating both diagnostic and therapeutic potential in a single platform [10]. Their biocompatibility and tunable size further enhance their suitability for in vivo applications, making them one of the most promising nanomaterials in cancer theranostics. Incorporating stimuli-responsive components into these systems is especially attractive, as it enables drug release in response to specific environmental triggers. Among various stimuli, pH is particularly relevant due to the acidic tumor microenvironment (pH 6.5–7.0), which contrasts with normal physiological conditions (pH 7.4). In addition, once internalized by cells, nanocarriers encounter the progressively acidifying endosomal-lysosomal pathway (pH 4.0–6.0), which can also promote drug release [15,16,17]. Nanocarriers that remain stable at physiological pH but undergo accelerated drug release under acidic conditions can therefore improve the therapeutic index of anticancer agents such as DOX [18,19,20].

Chitosan, a naturally occurring polysaccharide, and its derivatives have gained widespread attention as nanocarrier coatings owing to their biodegradability, biocompatibility, mucoadhesiveness, and cationic nature, which facilitates electrostatic interactions with negatively charged cell membranes [21,22]. Among them, N-[(2-hydroxy-3-trimethylammonium)propyl]chitosan chloride (HTCC) is particularly attractive because the presence of quaternary ammonium groups ensures solubility over a broad pH range and improves colloidal stability and cellular uptake [23,24]. HTCC can be further chemically modified to ensure robust anchoring to inorganic surfaces, enabling the formation of robust core–shell nanostructures [25,26,27]. This is commonly achieved through silane coupling agents, which create covalent links between the polymer and silica-based shells. Among the widely used silanes, (3-isocyanatopropyl)triethoxysilane (ICPTES) and (3-glycidyloxypropyl)trimethoxysilane (GPTMS) are particularly relevant for coupling chitosan derivatives to silica networks [25,28]. ICPTES isocyanate group readily reacts with nucleophilic groups such as HTCC hydroxyl or amino functionalities forming urethane linkages, while its triethoxysilane moiety participates in siloxane condensation with silica precursors [29]. This dual reactivity enables the formation of stable hybrid organic–inorganic interfaces. In contrast, GPTMS epoxide ring undergoes nucleophilic attack by HTCC amino groups, leading to covalent bond formation via ring-opening reactions [30]. This chemistry allows GPTMS to act as a versatile crosslinker, enhancing both mechanical strength and chemical stability of the resulting hybrid shell.

Despite the increasing interest in chitosan-based nanocarriers, systematic comparisons of silane functionalization strategies for quaternary chitosan on magnetic silica nanostructures remain scarce. Such functionalization is expected to strongly affect coating stability, drug loading capacity, and pH-responsive release. In this study, we develop and compare two pH-responsive magnetic nanocarriers based on HTCC functionalized with either ICPTES or GPTMS to enhance its anchoring on Fe_3_O_4_ cores. These nanocarriers were comprehensively characterized to evaluate their structural integrity, colloidal stability, DOX encapsulation efficiency, and release kinetics under physiologically relevant pH conditions. Additionally, we evaluate the cytotoxicity of DOX-loaded nanocarriers in MCF-7 breast cancer cells to assess their therapeutic potential.

## 2. Materials and Methods

### 2.1. Materials and Instruments

All reagents and solvents employed were of analytical purity and utilized as obtained, without any additional refinement. Doxorubicin hydrochloride (DOX, C_27_H_29_NO_11_·HCl, >99%) was sourced from Cayman Chemical (Ann Arbor, MI, USA). Phosphate-buffered saline (PBS) powder at pH 7.4, tetraethyl orthosilicate (TEOS, (Si(OC_2_H_5_)_4_, >99%), sodium citrate tribasic dihydrate (Na_3_C_6_H_5_O_7_·2H_2_O, >99%), 3-(triethoxysilyl)propyl isocyanate (ICPTES, (C_2_H_5_O)_3_Si(CH_2_)_3_NCO, 95%), 3-(glycidyloxypropyl)trimethoxysilane (GPTMS, C_9_H_20_O_5_Si, >98%) were purchased from Sigma-Aldrich (St. Louis, MO, USA). Sodium phosphate dibasic dodecahydrate (Na_2_HPO_4_·12H_2_O) was obtained from Panreac (Barcelona, Spain), while sodium phosphate anhydrous (Na_3_PO_4_) was purchased from Merk (Darmstadt, Germany). Ethanol (CH_3_CH_2_OH, >99%) was supplied by Chemlab (Zedelgem, Belgium). Methanol (CH_3_OH, >99%), ammonium hydroxide solution (NH_4_OH), and hydrochloric acid (HCl, 37%) were purchased from VWR (Radnor, PA, USA). N,N-dimethylformamide (HCON(CH_3_)_2_, >99%) was obtained from Fluka (Buchs, Switzerland), and potassium hydroxide (KOH, >86%) was acquired from LabChem (Zelienople, PA, USA). N-(2-hydroxypropyl-3-trimethylammonium chitosan chloride (HTCC, (C_12_H_25_N_2_O_5_Cl)_n_(C_8_H_13_NO_5_)_m_, degree of substitution ≥ 0.6) was purchased from CD Bioparticles-Drug Delivery (Shirley, NY, USA). All aqueous solutions were prepared with ultrapure water (18.2 MΩ·cm) produced by a Milli-Q purification system (Millipore, Burlington, MA, USA).

Cell culture reagents included RPMI 1640 medium (catalogue #R6504), L-glutamine, NaHCO_3_, trypsin–EDTA (ethylenediamine tetra acetic acid) solution, phosphate-buffered saline (PBS), NADH (reduced nicotinamide adenine dinucleotide), and sodium pyruvate were all obtained from Sigma-Aldrich. Fetal bovine serum (FBS), and penicillin/streptomycin/amphotericin B (antibiotics) solution purchased from PAN-Biotech™ (Aidenbach, Germany). Culture dishes and plates were purchased from TPP^®^ (Trasadingen, Switzerland).

High-resolution transmission electron microscopy (TEM) images were captured using a JEOL 2200FS instrument (Tokyo, Japan) operating at an accelerating voltage of 200 kV. Scanning electron microscopy (SEM) was carried out on a Hitachi SU8600 system (Tokyo, Japan) at 30 kV. For these analyses, powdered samples were suspended in ultrapure water, and an aliquot of 10 µL was deposited onto carbon-coated copper grids, followed by air drying. Particle dimensions were evaluated by measuring the diameters of 100 individual particles using ImageJ softwareversion 1.5.3 (NIH, Bethesda, MD, , USA). The elemental composition of the powders was assessed using a Leco Truspec-Micro CHNS 630-200-200 analyzer (St. Joseph, MI, USA). The X-ray diffraction (XRD) measurements were carried out on a PANalytical Empyrean X-ray diffractometer (Malvern, UK) equipped with the Cu Kα monochromatic source at 45 kV and 40 mA. Fourier transform infrared (FTIR) spectra were recorded with a Bruker Optics Tensor 27 spectrometer (Ettlingen, Germany) coupled to a horizontal attenuated total reflectance (ATR) cell, using 256 scans at a resolution of 4 cm^−1^. Thermal stability was assessed by thermogravimetric analysis (TGA) using a Hitachi STA300 system (Tokyo, Japan), heating the samples from 25 °C to 900 °C at a rate of 10 °C/min, in air. The nanoparticle surface charge was determined by measuring the zeta potential via electrophoretic light scattering in aqueous suspensions using a Zetasizer Nano ZS (Malvern Instruments, Malvern, UK). DOX concentrations were quantified by absorbance measurements with a SpectraMax^®^ ABS Plus microplate reader (Molecular Devices, (San Jose, CA, USA)).

### 2.2. Synthesis of Magnetic Nanocarriers

The magnetic nanocarriers were fabricated through a three-step synthetic process: (i) synthesis of the magnetic core (magnetite–Fe_3_O_4_); (ii) functionalization of HTCC using two distinct alkoxysilane coupling agents (ICPTES and GPTMS); and (iii) coating of the magnetic nanoparticles with a silica shell incorporating the modified HTCC.

Magnetite (Fe_3_O_4_) nanoparticles (NPs) were synthesized via the co-precipitation of ferric (Fe^3+^) and ferrous (Fe^2+^) ions, followed by surface stabilization with citrate ions [20,31]. Specifically, 4.43 g of FeCl_3_·6H_2_O and 1.625 g of FeCl_2_·4H_2_O were dissolved in 190 mL of deoxygenated ultrapure water at room temperature under a nitrogen atmosphere, with continuous mechanical stirring at 650 rpm. To initiate the reaction, 10 mL of ammonium hydroxide solution was added, and the mixture was stirred for 10 min. The resulting Fe_3_O_4_ NPs were magnetically separated using a NdFeB magnet and rinsed five times with 50 mL of distilled water. Before coating, the Fe_3_O_4_ NPs were stabilized with citrate ions to prevent aggregation. For this purpose, the NPs were washed twice with 100 mL of 2 M HNO_3_ and magnetically separated. Subsequently, the NPs were washed once with distilled water, dispersed in 200 mL of ultrapure water, and the pH adjusted to 2.5 by adding a few drops of 1 M NaOH solution. Then, 5 mL of 0.5 M sodium citrate tribasic dihydrate was added to the suspension, which was stirred mechanically for 1 h at room temperature. Finally, 50 mL of acetone was used to induce the precipitation the Fe_3_O_4_ NPs, which were then magnetically collected, washed with ethanol and distilled water, and dried by solvent evaporation.

The quaternary chitosan derivative (HTCC) was functionalized using two alkoxysilane coupling agents, 3-isocyanatopropyltriethoxysilane (ICPTES) and 3-(glycidoxypropyl)trimethoxysilane (GPTMS) [25,32]. In brief, HTCC-ICPTES and HTCC-GPTMS were synthesized by reacting dry HTCC (1 g) with dry dimethylformamide (DMF, 13 mL) and either ICPTES (5.2 mmol) or GPTMS (5.2 mmol). The reactions were conducted at 100 °C under a dry nitrogen atmosphere with continuous stirring at 500 rpm for 24 h. After cooling to room temperature, the resulting HTCC-ICPTES and HTCC-GPTMS products were thoroughly washed with anhydrous methanol and anhydrous ethanol. Finally, the functionalized materials were dried by solvent evaporation.

For the coating step, 100 mg of Fe_3_O_4_ NPs were dispersed in 18 mL of ultrapure water, sonicated for 10 min, and kept in an ice bath to prevent overheating [20,31]. Then, the modified biopolymer (HTCC-ICPTES or HTCC-GPTMS, 0.3 g), TEOS (0.4 mL), and ethanol (3 mL) were added to the dispersion, followed by triethylamine (0.1 mL) as a catalyst. The mixture was then sonicated for 15 min while maintained in the ice bath. The resulting coated nanoparticles—designated as MNP-HTCC1 for ICPTES and MNP-HTCC2 for GPTMS—were magnetically separated, thoroughly washed with acetone and ethanol, and dried at room temperature.

### 2.3. Preparation of DOX-Loaded Magnetic Nanocarriers

Doxorubicin (DOX) was selected as a model chemotherapeutic agent to evaluate the drug-loading efficiency and release behavior of the synthesized nanocarriers. The DOX solutions were prepared in PBS buffer at pH 9.5. To prepare the DOX-loaded formulations, 2.5 mg of either MNP-HTCC1 or MNP-HTCC2 were dispersed in 2 mL of aqueous DOX solution at concentrations ranging from 42 to 225 µg/mL. The mixtures were gently stirred at 30 rpm under light-protected conditions at room temperature for 24 h to ensure equilibrium adsorption. Following incubation, the DOX-loaded magnetic nanocarriers were magnetically separated using a bench NdFeB magnet. To determine the loading efficiency, the supernatant was collected, and the residual DOX concentration was quantified using a previously established calibration curve (Figure S1, Supplementary Information) by measuring the absorbance at 498 nm with a UV–VIS spectrophotometer. The loading efficiency of DOX in MNP-HTCC1 and MNP-HTCC2 and the nanoparticle drug-loading capacity were calculated using Equations (1) and (2), respectively:

(1) $$Loading\;efficiency\;(\%)=\frac{C_{initial}-C_{free}}{C_{initial}}\times 100$$

(2) $$Nanoparticle\;capacity\;(µg\;DOX/mg\;NP)=\frac{m_{loaded\;DOX}\;(µg)}{m_{NPs}\;(mg)}$$

where C_initial_ is the initial concentration of DOX (µg/mL), C_free_ is the concentration of free (unloaded) DOX in the supernatant after loading (µg/mL), m_loaded DOX_ is the mass of DOX (µg) actually loaded into the nanoparticles, and m_NPs_ is total weight of nanoparticles (mg) used in the drug loading process.

### 2.4. Release of DOX from Magnetic Nanocarriers at Different pH Conditions

To evaluate the pH-responsive release behavior of the DOX-loaded magnetic nanocarriers, 2.5 mg of each formulation (DOX-MNP-HTCC1 and DOX-MNP-HTCC2) were separately dispersed in 10 mL of PBS buffer at pH 4.2, 5.0, and 7.4. The suspensions were incubated at 37 °C under light-protected conditions with gentle shaking (30 rpm). At predetermined time intervals (10 min, 20 min, 30 min, 1 h, 2 h, 3 h, 4 h, 5 h, 24 h, 48 h and 72 h), 0.2 mL of the release medium was withdrawn for immediate analysis using a UV-VIS microplate reader for DOX content, then returned to the original vial to maintain sink conditions. Before sampling, the nanocarriers were magnetically separated to the bottom of the vial to ensure that only the drug released into the supernatant was measured, thereby avoiding interference from the magnetic nanoparticles. The amount of DOX released was calculated using Equation (3):

(3) $$Release\;(\%)=\frac{DOX\;{mass}_{release\;at\;t}}{DOX\;{mass}_{initial}}\times 100$$

where DOX mass*_release_* is the total mass of DOX release at time *t* (µg) and DOX mass_i_*_nitial_* is the total mass of DOX (µg) loaded in the MNPs used in the release experiment. A separate calibration curve was established for each pH condition to accurately convert absorbance values into DOX concentration (Figure S2, Supplementary Information).

### 2.5. Cell Culture and Treatment

The biological assays were designed to provide proof-of-concept evidence of the functional cytotoxic activity of the DOX-loaded nanocarriers, rather than to perform an extensive biological validation across multiple cancer phenotypes. The MCF-7 breast cancer cell line (an estrogen receptor–positive human breast adenocarcinoma line; ATCC HTB-22; passages 20–23) was cultured in a humidified atmosphere of 5% CO_2_ and 95% air. Cells were grown in RPMI-1640 medium supplemented with 2 mM L-glutamine, 10 mM sodium bicarbonate, 10% heat-inactivated fetal bovine serum (FBS), and 1% antibiotic/antimycotic solution. The medium was refreshed every 2–3 days, and cultures were subcultured weekly. For passaging, cells were detached enzymatically using 0.05% trypsin–EDTA for 5 min at 37 °C, then split at a 1:4 ratio into plastic culture dishes (21 cm^2^; ∅ 60 mm).

For the cell viability assays, MCF-7 cells were seeded in 96-well plates and grown to 80–90% confluence. Treatments were applied in serum-free medium for 24 h. A 5 mM DOX stock solution was prepared in ultrapure water and diluted in culture medium to final concentrations ranging from 1 to 50 µM. The DOX-loaded MNPs were dispersed in culture medium by sonication for 5 s, then diluted to achieve equivalent DOX concentrations based on the 24-h release profile. The blank (non-loaded) MNPs were prepared and diluted using the same procedure to match the nanoparticle concentrations used in the DOX-loaded MNP treatments.

### 2.6. Cytotoxicity Studies

Cell viability in MCF-7 cells was assessed by measuring extracellular lactate dehydrogenase (LDH) activity, which is an indicator of loss of plasma membrane integrity. LDH release into the culture medium was quantified spectrophotometrically by monitoring the decrease in NADH absorbance at 340 nm during the pyruvate-to-lactate conversion, as previously described [33]. The results were expressed as the percentage of extracellular LDH activity relative to control cells. For EC_50_ determination, LDH activity was calculated as the percentage of extracellular activity relative to total cellular LDH activity. Total LDH activity was obtained by lysing control cells with 0.1% (*v*/*v*) Triton X-100 for 30 min at 37 °C.

All data are presented as mean ± standard error of the mean (SEM), with n representing replicate measurements from three independent experiments. Statistical comparisons between treated groups and controls were performed using Student’s *t*-test (*p* < 0.05 considered statistically significant) in Microsoft Excel 365 (Version 2501; Microsoft, Redmond, WA, USA). GraphPad Prism 9 (GraphPad Software, San Diego, CA, USA) was used for EC_50_ calculations and graphical data presentation.

## 3. Results

### 3.1. Physicochemical Characterization of HTCC-Functionalized Magnetic Nanocarriers

**Figure 1:**
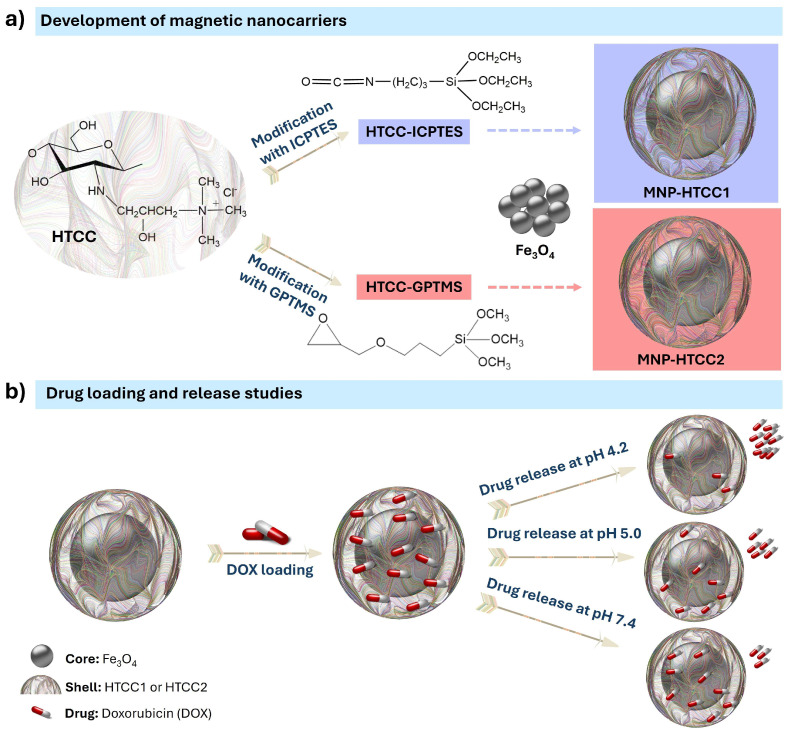
Schematic illustration of (**a**) the preparation of magnetic nanocarriers MNP-HTCC1 and MNP-HTCC2, showing the chemical structure of the quaternary chitosan (HTCC) modified with distinct alkoxysilane agents (ICPTES: 3-(triethoxysilyl)propyl isocyanate; GPTMS: 3-(glycidyloxypropyl)trimethoxysilane) and (**b**) the loading of doxorubicin (DOX) and its release under different pH conditions (4.2, 5.0, and 7.4). Doxorubicin is incorporated within the HTCC–silica shell through electrostatic interactions and is released in a pH-dependent manner.

Two distinct magnetic nanocarriers, MNP-HTCC1 and MNP-HTCC2, were prepared through the synthesis of Fe_3_O_4_ magnetic cores, the functionalization of quaternary chitosan (HTCC) with different alkoxysilane agents (ICPTES and GPTMS), and subsequent coating of the magnetic cores with a siliceous shell incorporating the modified HTCC (Figure 1a). As schematically illustrated in Figure 1a, the Fe_3_O_4_ nanoparticles serve as magnetic cores, while the HTCC-ICPTES and HTCC-GPTMS conjugates form the external HTCC-silica hybrid functional shell responsible for drug loading and pH-responsive release. Doxorubicin (DOX) was subsequently loaded through electrostatic interactions and hydrogen bonding with the HTCC-rich shell, rather than with the magnetic core, which remains physically inaccessible to the drug molecules. The drug release behavior was assessed under different pH conditions (4.2, 5.0, and 7.4) to mimic various physiological environments (Figure 1b). The acidic conditions (pH 4.2 and 5.0) were selected to model the microenvironments typically encountered after nanoparticle internalization, such as endosomes and lysosomes, where protonation of the HTCC shell can weaken electrostatic interactions with DOX and promote its release. In contrast, pH 7.4 represents the bloodstream and healthy tissue environment, where stronger interactions are maintained and a slower release profile is anticipated. Examining DOX release under these controlled conditions provides a basis for understanding the stimuli-responsive behavior of the HTCC shell and evaluating the suitability of the designed nanocarriers for targeted and controlled anticancer drug delivery.

**Figure 2:**
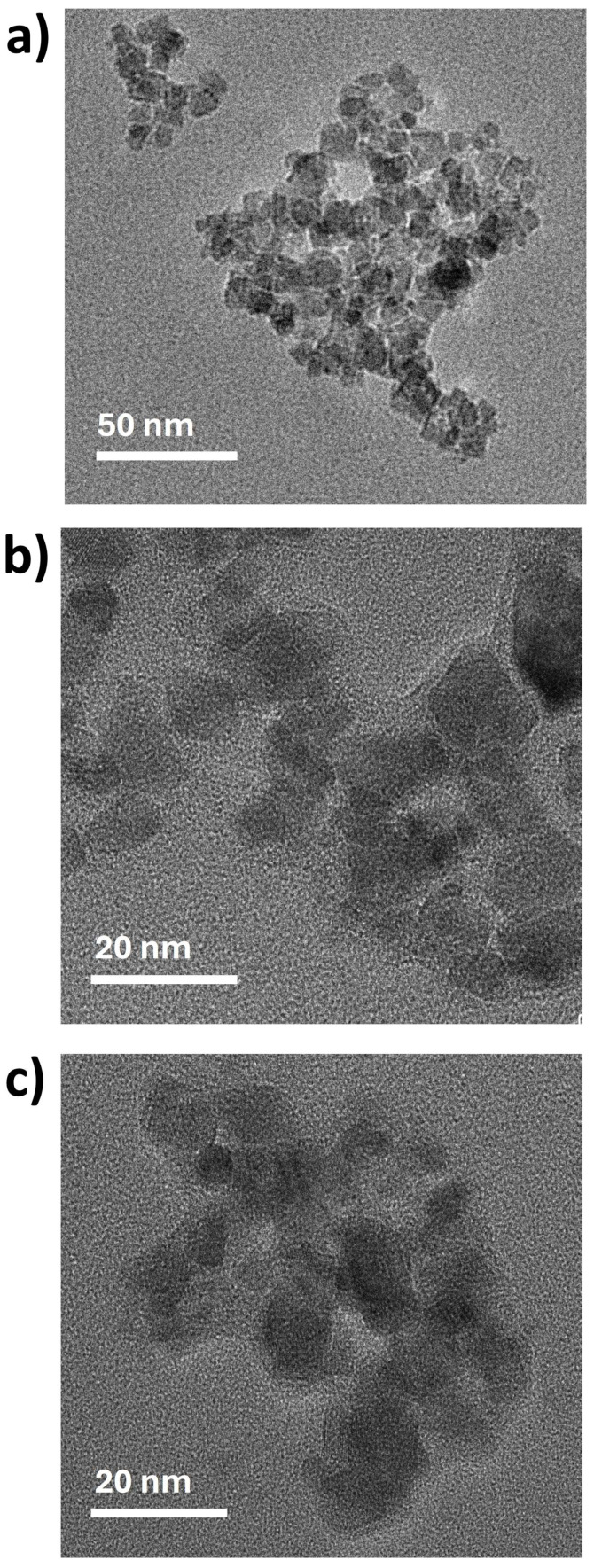
TEM images of (**a**) Fe_3_O_4_, (**b**) MNP-HTCC1 and (**c**) MNP-HTCC2.

The morphology and size of the magnetic nanocarriers were examined using transmission and scanning electron microscopy (TEM/SEM), as shown in Figure 2 and Table 1. TEM images reveal that the bare Fe_3_O_4_ NPs were predominantly spheroidal, with some irregularly shaped particles (Figure 2a), and presented an average size of 11.5 nm. The coated magnetic nanocarriers exhibit a core–shell morphology, with average diameters of 14.5 nm and 12.6 nm for MNP-HTCC1 (Figure 2b) and MNP-HTCC2 (Figure 2c), respectively. The HTCC coating surrounding the Fe_3_O_4_ cores could be distinguished as lighter contrast regions enveloping the darker magnetic cores. Complementary scanning electron microscopy (SEM) and bright-field scanning transmission electron microscopy (BF-STEM) images of Fe_3_O_4_, MNP-HTCC1, and MNP-HTCC2 nanoparticles (Figure S3, Supplementary Information), further confirmed the presence of the coating, validating the successful surface modification of the magnetic cores. Overall, TEM and SEM analyses confirmed the formation of well-defined core–shell nanostructures with nanoscale dimensions suitable for biomedical applications.

The crystalline structure and phase composition of the nanoparticles were examined by X-ray diffraction (XRD). The main diffraction peaks of magnetite (Fe_3_O_4_) were consistent with its characteristic inverse spinel structure (Figure S4, Supplementary Information). The diffraction patterns display the prominent reflections at 2θ ≈ 30.1°, 35.5°, 43.2°, 53.5°, 57.1°, and 62.7°, which correspond to the (220), (311), (400), (422), (511), and (440) planes of magnetite, respectively [34]. For the MNP-HTCC1 and MNP-HTCC2, the same diffraction pattern was observed with slight reductions in peak intensity, which can be attributed to the amorphous nature of the siliceous polymer shell surrounding the crystalline magnetic core [35].

In addition to the structural characterization, the magnetic properties of the Fe_3_O_4_ core are expected to be consistent with those previously reported by Nogueira et al. [20], as the same synthetic procedure was employed for the preparation the magnetite nanoparticles. While the surface coating differs, the Fe_3_O_4_ cores exhibited an average diameter of 10 ± 2.5 nm, which is comparable to the core sizes obtained for MNP-HTCC1 and MNP-HTCC2 (11 ± 5.1 nm). As observed by Nogueira et al. [20], both bare and surface-modified Fe_3_O_4_ nanoparticles displayed superparamagnetic-like behavior, attributed to the small size of the magnetic cores. The bare Fe_3_O_4_ nanoparticles showed a saturation magnetization of approximately 25 emu/gFe_3_O_4_ at 300 K. Considering that the polymeric shell surrounding the magnetic cores in MNP-HTCC1 and MNP-HTCC2 is only a few nanometers thick, the coated particles are expected to exhibit magnetic saturation values nearly identical to those of the uncoated Fe_3_O_4_ nanoparticles, with only a slight decrease arising from the presence of the non-magnetic shell. The elemental composition was assessed by CHN microanalysis to confirm the presence of HTCC in the coated nanocarriers (Table 1). As expected, the bare Fe_3_O_4_ nanoparticles exhibited only trace amounts of carbon and nitrogen, reflecting their inorganic composition. In contrast, MNP-HTCC1 and MNP-HTCC2 displayed significantly higher carbon contents (11–15 wt.%) and nitrogen levels of 2–3 wt.%, confirming the successful attachment of the organic HTCC shell to the magnetic cores. For comparison, the HTCC derivatives (HTCC-ICPTES and HTCC-GPTMS) showed even higher carbon (42 wt.%) and nitrogen (8 wt.%) contents, consistent with their organic nature. The similar elemental composition of HTCC-ICPTES and HTCC-GPTMS indicates that both silane functionalization routes led to a comparable degree of substitution and preserved the HTCC backbone.

**Figure 3:**
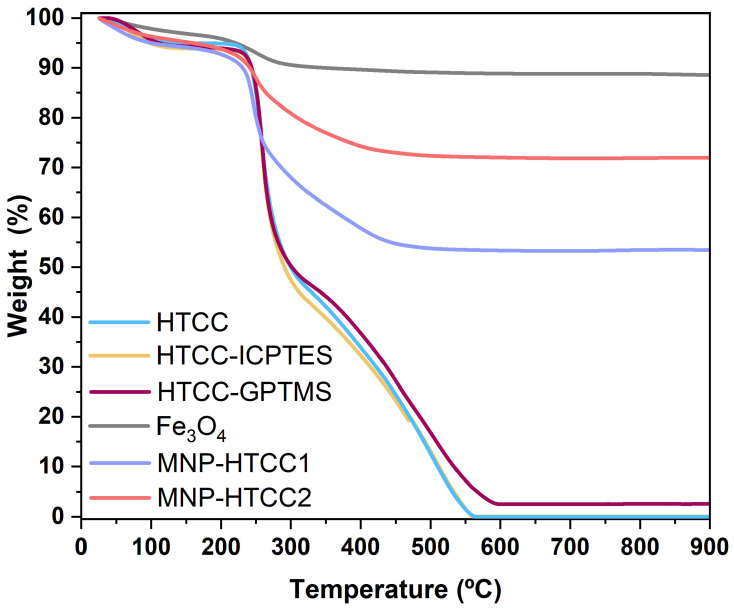
Thermogravimetric analysis (TGA) curves for HTCC, HTCC-ICPTES, HTCC-GPTMS, bare magnetite nanoparticles (Fe_3_O_4_), MNP-HTCC1, and MNP-HTCC2.

Thermogravimetric analysis (TGA) was conducted to evaluate the thermal stability and composition of the magnetic nanocarriers, and the corresponding weight loss profiles are shown in Figure 3. The bare Fe_3_O_4_ nanoparticles exhibit minimal weight loss (<12 wt. %), consistent with the removal of surface-adsorbed water and citrate species. In contrast, MNP-HTCC1 and MNP-HTCC2 showed pronounced mass losses between 200 °C and 500 °C due to the thermal decomposition of the HTCC coating, thereby confirming successful surface modification of the magnetic cores. For the pristine HTCC, a small initial weight loss (25–100 °C) was observed due to moisture desorption [36] followed by a major degradation step between 100 °C and 350 °C, corresponding to the decomposition of the HTCC polymer backbone and the cleavage of quaternary trimethyl ammonium (–N(CH_3_)_3_^+^) groups [35]. Between 350 °C and 550 °C, a secondary degradation phase may be observed, where the remaining volatile components (such as short-chain degradation products or fragments) undergo additional decomposition [36,37]. Finally, from 550 °C to 900 °C, the mass stabilizes, corresponding to the formation of an inorganic residue. The TGA profiles of HTCC-ICPTES and HTCC-GPTMS closely resembled that of pristine HTCC, indicating that the incorporation of ICPTES or GPTMS did not significantly alter the intrinsic thermal stability of the polymer. In contrast, the hybrid nanocarriers exhibited distinct behaviors: MNP-HTCC1 showed a higher total organic weight loss (~47%) than MNP-HTCC2 (~28%), reflected in the lower residual mass fractions of 53% and 72%, respectively. This trend is consistent with the elemental microanalysis data (Table 1), which revealed higher carbon and nitrogen contents for MNP-HTCC1 (15.4% C, 3.2% N) than for MNP-HTCC2 (11.4% C, 2.3% N). These differences likely arise from variations in surface modification or the degree of HTCC coating, which influence the thermal stability and decomposition behavior of the magnetic nanocarriers.

**Figure 4:**
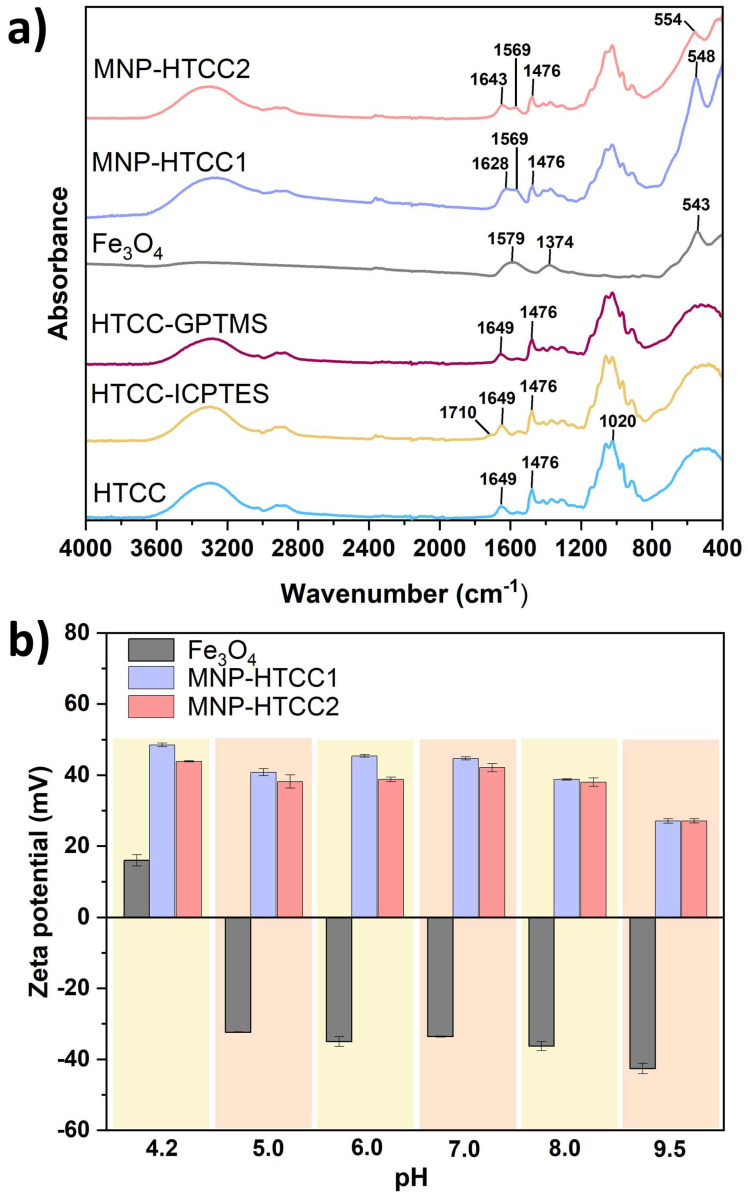
(**a**) FTIR spectra of HTCC, HTCC-ICPTES, HTCC-GPTMS, Fe_3_O_4_, MNP-HTCC1 and MNP-HTCC2 and (**b**) zeta potential values of Fe_3_O_4_, MNP-HTCC1 and MNP-HTCC2 as function of pH in the range 4.2–9.5.

The presence of specific functional groups in the synthesized materials was confirmed by ATR-FTIR spectroscopy, as shown in Figure 4a. The spectrum of HTCC exhibits characteristic absorption bands that differentiate it from conventional chitosan. Specifically, a sharp band at 1479 cm^−1^ corresponds to the C–H asymmetric bending vibration of the trimethylammonium group, confirming the presence of quaternary ammonium functionalities [38]. Additionally, a prominent peak at 1649 cm^−1^ was attributed to the secondary amine vibrations in HTCC [38]. Both vibrational bands were retained in the spectra of the modified HTCC (HTCC-ICPTES and HTCC-GPTMS) and magnetic nanocarriers (MNP-HTCC1 and MNP-HTCC2), indicating that the HTCC chemical backbone remained preserved after silane functionalization and nanoparticle coating. In HTCC-ICPTES, a new band appeared at 1710 cm^−1^, assigned to the C=O stretching of urethane groups, confirming covalent bonding between ICPTES and HTCC [29]. This confirms the covalent incorporation of ICPTES into HTCC via urethane linkages, enhancing the polymer’s structural stability and affinity for the inorganic phase. In contrast, no new intense absorption bands appeared for HTCC-GPTMS, consistent with previous reports in which epoxy-and silane-related signals (e.g., Si–O–Si at ~1080 cm^−1^, epoxy vibrations at ~910 cm^−1^) are often masked by the broad HTCC bands or occur at intensities below detection thresholds [39,40]. The FTIR spectra of bare Fe_3_O_4_ NPs displayed a strong band centered at 543 cm^−1^, assigned to the Fe–O stretching vibration [20]. This band is also evident in the magnetic nanocarriers but appears slightly shifted to 548 cm^−1^ for MNP-HTCC1 and 554 cm^−1^ for MNP-HTCC2, indicating successful coating of magnetite with the HTCC shells. Furthermore, two additional bands at 1374 cm^−1^ and 1579 cm^−1^ are observed in the Fe_3_O_4_ spectrum, corresponding to the stretching of –CO and the asymmetric stretching of –COO^−^ groups, respectively, arising from the citrate ions used to stabilize the colloidal magnetite NPs [20,31]. The overlapping of these citrate-related bands with HTCC vibrations resulted in a spectral profile that reflects the coexistence of organic and inorganic components in the magnetic nanocarriers.

The zeta potential values as a function of pH (Figure 4b) provided insights into surface charge characteristics and colloidal stability. Bare Fe_3_O_4_ nanoparticles exhibited strongly negative zeta potential values, ranging from −32.4 mV at acidic pH (5.0) to −42.6 mV at alkaline pH (9.5), consistent with the deprotonation of surface hydroxyl groups and the presence of adsorbed citrate ions used for stabilization. This pronounced negative charge promotes electrostatic repulsion between particles, enhancing dispersion but limiting interaction with negatively charged biological structures. In contrast, both MNP-HTCC1 and MNP-HTCC2 displayed highly positive surface charges over the pH range 4.2–9.5, ranging from +45.4 mV at acidic pH to +27.1 mV at near-neutral to alkaline pH, reflecting the strong influence of cationic quaternary ammonium groups (–N(CH_3_)_3_^+^) introduced via HTCC modification [37]. The strong positive surface charge of the HTCC-coated nanocarriers promotes electrostatic stabilization and prevents aggregation even under physiological conditions, enhancing their colloidal stability. This cationic character also favors interactions with negatively charged biomolecules such as proteins, nucleic acids, and cell membranes, thereby improving cellular uptake and potential for targeted drug delivery [22,41]. The persistence of high positive zeta potential across a broad pH range demonstrates the robustness of the surface functionalization against charge reversal. The slightly higher zeta potential values observed for MNP-HTCC1 may result from a greater HTCC content and a more homogeneous coating compared to MNP-HTCC2, which may contribute to its superior stability and biointeraction capacity. Overall, the tunable surface charge achieved through silane-assisted HTCC functionalization represents a key design parameter for optimizing nanocarrier performance in biomedical applications.

### 3.2. Doxorubicin Loading and Release Studies

**Figure 5:**
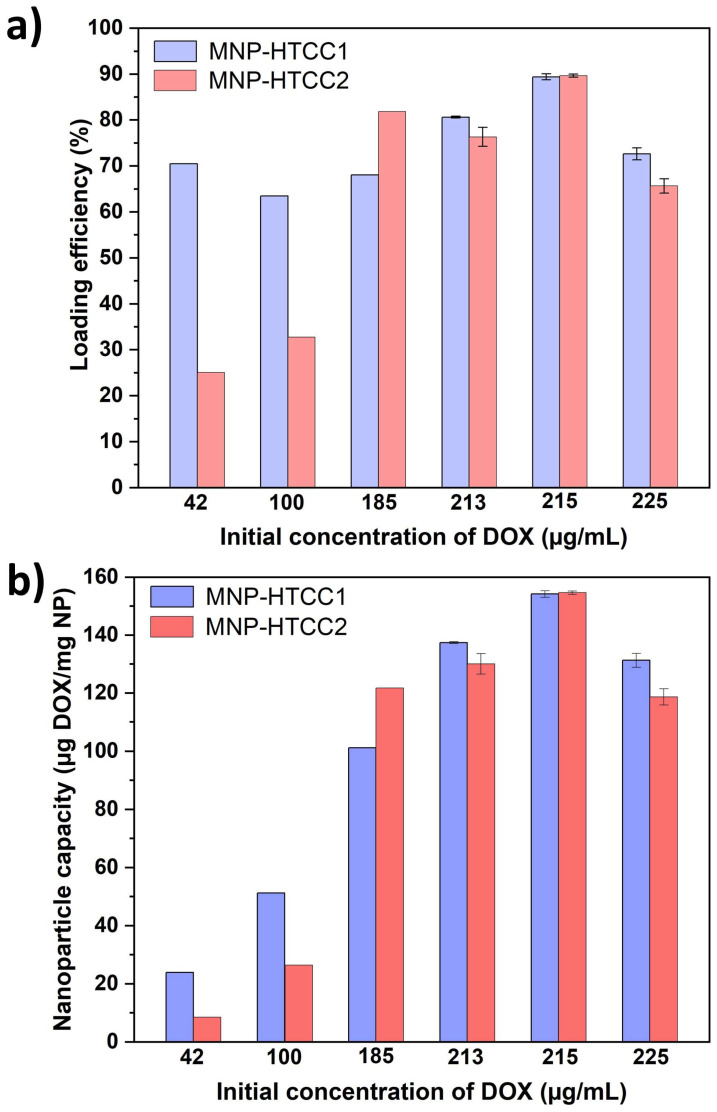
(**a**) Loading efficiency (%) of DOX and (**b**) nanoparticle capacity (ug DOX/mg NP) for MNP-HTCC1 and MNP-HTCC2 at pH 9.5 in PBS. The loading experiments were performed at varying initial DOX concentrations ranging from 42 to 225 µg/mL.

Doxorubicin (DOX), a clinically relevant anticancer drug, was selected as a model compound to evaluate the loading performance and pH-responsiveness of the synthesized magnetic nanocarriers. Drug loading was conducted under alkaline conditions (pH 9.5 in PBS), which favor the electrostatic adsorption of anionic DOX species (Figure S5, Supplementary Information) onto the cationic HTCC-based nanocarrier surface. As shown in Figure 5a, MNP-HTCC1 exhibited consistently higher loading efficiency (63–90%) across the tested DOX concentrations, whereas MNP-HTCC2 displayed lower efficiency (25–33%) at low DOX concentrations (42–100 µg/mL), but increased sharply to 90% at 215 µg/mL. This behavior indicates that MNP-HTCC1 ensures efficient and stable DOX loading over a broad concentration range, while MNP-HTCC2 becomes effective only at higher drug levels. The superior and more consistent loading performance of MNP-HTCC1 is likely due to its higher surface coverage with HTCC that provides a greater number of amine and cationic binding sites (–NH_2_ and –N(CH_3_)_3_^+^) for electrostatic and H-bond interactions with the negatively charged DOX molecules, even at low concentrations, as supported by elemental, zeta potential and TGA data. Conversely, the lower loading efficiency of MNP-HTCC2 at low DOX concentrations can be attributed to a less homogeneous HTCC coating, providing fewer accessible binding sites; at higher concentrations, diffusion-driven compensates for this limitation, explaining the sudden rise in loading efficiency near saturation.

The drug-loading capacity (expressed as µg of DOX per mg of magnetic nanocarrier, Figure 5b), followed a similar trend. For MNP-HTCC1, the capacity increased steadily with the initial DOX concentration, from 23 µg/mg at 42 µg/mL to 154 µg/mg at 215 µg/mL. MNP-HTCC2 showed a comparable maximum capacity (154 µg/mg at 215 µg/mL) but much lower values at low concentrations (8 µg/mg at 42 µg/mL). Both systems reached saturation at 215 µg/mL, beyond which capacity slightly decreased, likely due to surface saturation or mild desorption effects. Throughout the concentration range, MNP-HTCC1 generally maintained higher capacities at lower DOX concentrations, suggesting its stronger drug–surface affinity.

**Figure 6:**
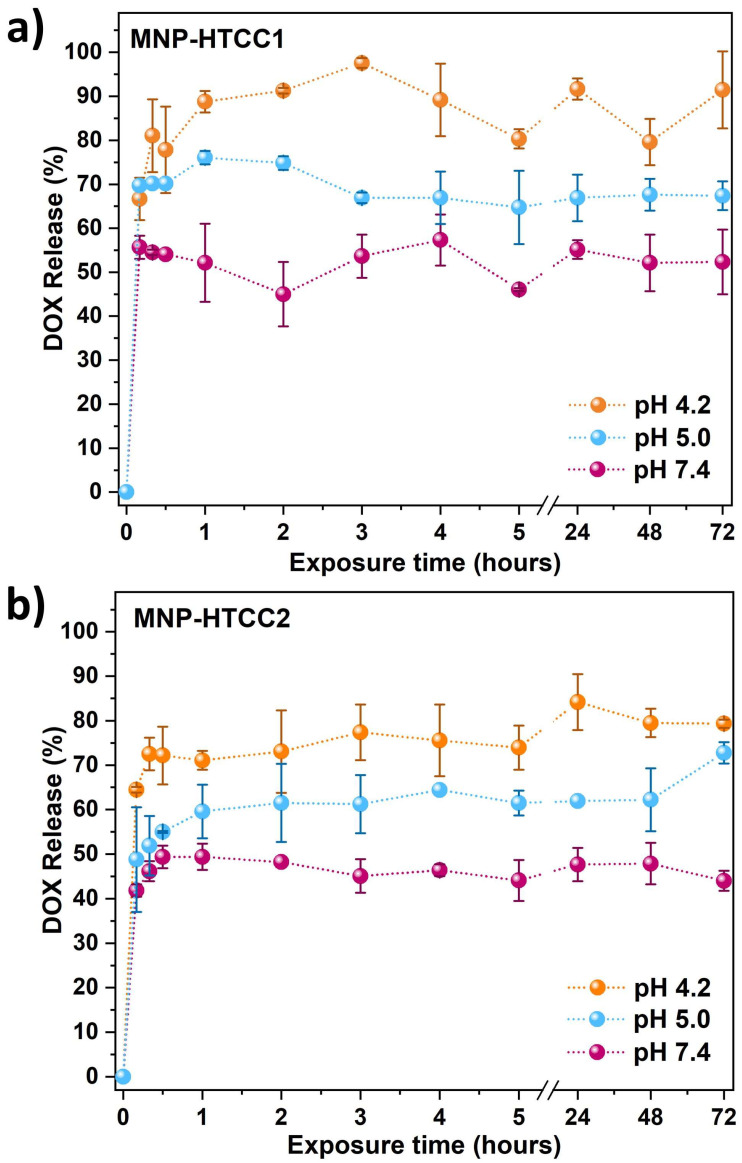
Release profile of doxorubicin (DOX) from (**a**) DOX-loaded MNP-HTCC1 and (**b**) DOX-loaded MNP-HTCC2 at 37 °C under pH 4.2, 5.0 and 7.0 over a period of 72 h. Both formulations had a loading capacity of 13.4 wt.%.

Based on their loading performance, MNP-HTCC1 and MNP-HTCC2 were subsequently evaluated for pH-responsive DOX release under physiologically relevant conditions (pH 4.2, 5.0, and 7.4) to mimic lysosomal, endosomal, and extracellular environments [20,42] (Figure 6). Both systems exhibited clear pH-dependent release behavior, with faster drug release under acidic conditions. This trend is consistent with the acid–base properties of doxorubicin (Figure S5, Supplementary Information): at pH values below 8, DOX becomes progressively protonated and predominantly exists as cationic species, which weakens its electrostatic interactions with the positively charged HTCC coating and facilitates its diffusion into the release medium. As a result, both nanocarriers released DOX more readily at pH 4.2–5.0, with MNP-HTCC1 generally showing higher release than MNP-HTCC2.

At pH 4.2, both nanocarriers released about 77% of DOX within the first 30 min, followed by a sustained phase reaching 90% for MNP-HTCC1 and 79% for MNP-HTCC2 after 72 h. Such a high release rate in acidic conditions is particularly advantageous for intracellular drug delivery, where rapid drug availability can enhance cytotoxic activity against cancer cells. This pronounced acid-triggered release reflects the combined contribution of DOX protonation and the responsiveness of the hybrid coating. Although the siliceous framework provides structural rigidity, the HTCC domains embedded within the hybrid HTCC–silica shell remains sensitive to pH variations: protonation of quaternary ammonium and residual amine groups increases hydration and local polymer mobility, loosening the hybrid network and facilitating drug diffusion [40]. These synergistic effects explain the accelerated release observed at pH 4.2, with MNP-HTCC1 exhibiting the highest cumulative release, consistent with its higher HTCC coverage and more homogeneous coating, which undergoes greater pH-driven relaxation and thus enables more efficient drug diffusion compared with MNP-HTCC2.

At pH 5.0, which mimics the environment of late endosomes [43], both magnetic nanocarriers exhibited intermediate release patterns, with 67–72% of DOX released, confirming their responsiveness to mildly acidic conditions relevant to intracellular drug delivery after cellular uptake and prior to lysosomal degradation, an essential characteristic for pH-triggered chemotherapy [42]. At physiological pH 7.4, both MNP-HTCC1 and MNP-HTCC2 exhibited moderate DOX release (43–55%), indicating partial drug retention under neutral conditions. Such controlled release minimizes premature leakage in circulation while maintaining a low baseline drug level that may improve systemic bioavailability.

The results showed that, regardless of pH, both magnetic nanocarriers exhibited an initial burst release of DOX within the first 30 min followed by sustained release over time. This rapid release can be therapeutically advantageous, and particularly beneficial for fast-proliferating cancers or for triggering synergistic effects with other treatments [2,44]. At the same time, the burst is moderate under physiological pH, reducing the risk of premature systemic toxicity during circulation [45]. Consequently, this initial release phase, followed by sustained drug release, provides a dual advantage: rapid therapeutic action at the target site while maintaining overall safety and prolonged efficacy. Collectively, these results confirm that both nanocarriers act as efficient pH-responsive delivery systems, with MNP-HTCC1 providing faster and more complete release, making it particularly suitable for targeted chemotherapy applications.

The DOX release data were fitted to the Weibull model (Equation (4)), which is a versatile empirical equation frequently employed to describe drug release kinetics from complex systems:

(4) $$F(t)=1-e^{\left(-\frac{{\left(t-T_{i}\right)}^{\beta }}{\alpha }\right)}$$

where F(t) is the fraction of drug released at time t, α is a scale parameter related to the release rate, Ti is the lag time before the onset of the drug release (in most cases zero), and β is the shape parameter that characterizes the curve as exponential (β = 1), S-shaped with upward curve followed by turning point (β > 1), or parabolic with higher initial slope after that consistent with the exponential (β < 1) [46].

The experimental data were fitted using non-linear regression by employing the method of least squares and the tool solver of the Excel software (version 2501). The goodness of the fitting was evaluated based on the analysis of the statistical parameter coefficient of determination (R^2^) and chi-square (χ^2^).

As shown in Table 2, all the studied formulations exhibited β values below 0.2 and good correlation coefficients (R^2^ > 0.95, χ^2^ < 0.04). The small β value indicates a diffusion-controlled release mechanism, consistent with Fickian transport through the siliceous HTCC-based matrix [47]. Overall, the α values increased with pH, consistent with slower DOX release under physiological conditions (pH 7.4) and faster diffusion in acidic media (pH 4.2–5.0). MNP-HTCC1 showed slightly lower α and β parameters than MNP-HTCC2, in agreement with a more homogeneous and higher-coverage HTCC coating, which enhances DOX–polymer interactions and favors a more sustained release.

The Korsmeyer–Peppas model, although commonly used for diffusion-controlled systems, was not applied in this study because it is generally valid only for the initial stage of release (typically up to 60% of the total drug released) [48]. Since several of the formulations investigated here exhibited cumulative release above this threshold, the use of the Weibull model was preferred, as it provides a more comprehensive description of the entire release profile. Nevertheless, previous work by Martín-Camacho et al. [47] demonstrated a strong correlation between the Weibull and Korsmeyer–Peppas parameters (β and n, respectively), supporting the validity of the mechanistic interpretation derived from the Weibull model.

Overall, the comparative analysis of MNP-HTCC1 and MNP-HTCC2 reveals a clear structure–function relationship, in which differences in silane-mediated surface chemistry and HTCC coverage directly influence drug loading efficiency and pH-responsive release behavior.

### 3.3. Cell Viability

**Figure 7:**
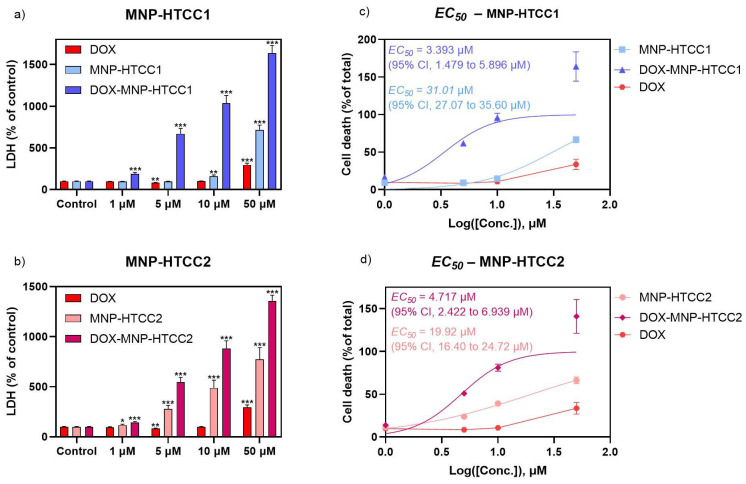
Effect of a 24 h exposure to (**a**): DOX, MNP-HTCC1 and DOX- MNP-HTCC1 (1–50 μM); and (**b**): DOX, MNP-HTCC2 and DOX- MNP-HTCC2 (1–50 μM) on MCF-7 cell viability by LDH (% of control) assay (*n* = 12). EC_50_ curves of (**c**) MNP-HTCC1 and DOX- MNP-HTCC1 and (**d**) MNP-HTCC2 and DOX- MNP-HTCC2, plotted together with DOX using LDH (% of total) results. Results are expressed as mean ± SEM. * indicates significant differences between sample and control at *p* < 0.05; ** *p* < 0.005; *** *p* < 0.0005 (Student’s *t* test).

To evaluate the cytotoxicity of the DOX delivery systems, MCF-7 cells were exposed for 24 h to free DOX, blank MNPs (MNP-HTCC), and DOX-loaded MNPs, followed by assessment of cell viability using the LDH assay (Figure 7). Accordingly, the biological data are presented to support the functional activity of the delivery system in a representative breast cancer model, without extrapolation to broader anticancer efficacy across distinct tumor phenotypes. Both blank and DOX-loaded MNPs exhibited a concentration-dependent cytotoxic effect, whereas free DOX showed minimal cytotoxicity at all concentrations except the highest (50 µM). The cytotoxicity observed for the blank nanocarriers is consistent with the cationic nature of HTCC-based systems and their strong electrostatic interactions with cell membranes under in vitro conditions. Importantly, it is clearly evident that, across all concentrations, the DOX-loaded MNPs demonstrated greater cytotoxicity than either the blank MNPs and free DOX. Furthermore, among the two formulations, MNP-HTCC1 displayed higher cytotoxic activity than MNP-HTCC2 in its DOX-loaded form (Figure 7). The comparative analysis of MNP-HTCC1 and MNP-HTCC2 reveals a clear structure–activity relationship, in which differences in silane-mediated surface chemistry and HTCC coverage govern drug loading, pH-responsive release, and the resulting cytotoxic response.

For all materials except free DOX, EC_50_ values were determined based on LDH activity, expressed as a percentage of total LDH release after cell lysis. MNP-HTCC1 (Figure 7c) exhibited an EC_50_ of 31.01 µM (95% CI, 27.07 to 35.60 µM), which decreased to 3.393 µM (95% CI, 1.479 to 5.896 µM) upon DOX loading. In comparison, MNP-HTCC2 (Figure 7d) showed an EC_50_ of 19.92 µM (95% CI, 19.40 to 24.72 µM), decreasing to 4.717 µM (95% CI, 2.422 to 6.939 µM) after DOX incorporation. Even though the EC_50_ of DOX was not calculated, Figure 7c,d also displays the LDH results of DOX, for comparison. Interestingly, among the two formulations, MNP-HTCC1 displayed higher cytotoxic potency than MNP-HTCC2 after DOX loading, despite the opposite trend in their unloaded forms, possibly due to the differences in surface modification between the two materials. Overall, these findings highlight the enhanced cytotoxic effect of the DOX-loaded MNP systems against MCF-7 cells, enabling a stronger therapeutic effect at lower doses, and emphasize the critical role of surface chemistry in modulating nanoparticle performance.

## 4. Conclusions

Two magnetic nanocarriers based on quaternary chitosan (HTCC) were successfully synthesized using distinct silane functionalization strategies: ICPTES for MNP-HTCC1 and GPTMS for MNP-HTCC2. MNP-HTCC1 displayed higher HTCC coverage, greater functional group density, and increased organic content, as confirmed by TGA, elemental analysis, and zeta potential measurements. These structural differences resulted in superior DOX-loading efficiency, higher capacity at low drug concentrations, and a more pronounced pH-responsive release profile. Both nanocarriers exhibited clear pH-dependent behavior, with an initial burst followed by sustained release, while retaining moderate amounts of DOX at physiological pH, which is advantageous for minimizing premature drug leakage. Under acidic conditions mimicking tumor and endo-lysosomal environments, MNP-HTCC1 reached nearly complete DOX release and consistently outperformed MNP-HTCC2 across all conditions, underscoring the strong impact of silane chemistry on drug–carrier interactions and release kinetics. In vitro cytotoxicity assays in MCF-7 cells showed that both DOX-loaded nanocarriers produced markedly higher cytotoxicity than their unloaded forms and free DOX, reflected in significantly lower EC_50_ values. MNP-HTCC1 showed the strongest response, consistent with its improved loading and release behavior. Overall, these results demonstrate a clear structure–activity relationship, in which differences in silane-mediated surface chemistry and HTCC coverage govern not only drug loading and pH-responsive release, but also the resulting biological response. MNP-HTCC1 therefore emerges as the most promising formulation, combining efficient loading with controlled, pH-triggered sustained release. These findings highlight the potential of HTCC-based magnetic nanocarriers as versatile platforms for smart anticancer drug delivery, with future applicability in magnetically assisted therapies and multifunctional theranostic systems. The biological evaluation included in this study was designed to support the functional performance and comparative assessment of the two nanocarrier systems, enabling the evaluation of how differences in silane-mediated surface chemistry translate into distinct cytotoxic responses within a representative breast cancer mode. While the observed cytotoxic effects support the potential of the DOX-loaded systems, further studies involving additional cancer cell lines with distinct molecular and phenotypic profiles, as well as non-cancerous cell models, will be required to extend, generalize, and assess the selectivity of these findings.

## Figures and Tables

**Table 1:** Quantification of carbon, hydrogen and nitrogen by elemental microanalysis.

Sample	C (%) ^1^	H (%) ^1^	N (%) ^1^	D (nm) ^2^
HTCC	36.8	8.49	7.00	-
HTCC-ICPTES (HTCC1)	42.1	7.89	8.27	-
HTCC-GPTMS (HTCC2)	42.6	8.05	8.38	-
Fe_3_O_4_	3.12	0.43	0.17	11.5 ± 5.1
MNP-HTCC1	15.4	2.52	3.22	14.5 ± 3.3
MNP-HTCC2	11.4	2.29	2.25	12.6 ± 3.9

^1^ Carbon, hydrogen and nitrogen content measured by elemental microanalysis; ^2^ Average particle diameter assessed by transmission electron microscopy.

**Table 2:** Kinetics parameters and goodness of the fits of DOX release from loaded magnetic nanocarriers using the Weibull model.

	MNP-HTCC1			MNP-HTCC2		
	pH 4.2	pH 5.0	pH 7.4	pH 4.2	pH 5.0	pH 7.4
α (min^−β^)	1.073	0.8047	1.3766	1.024	1.497	1.6784
β	0.1594	0.001	0.0131	0.0667	0.0558	0.0105
R^2^	0.9589	0.9867	0.9608	0.9878	0.9689	0.9760
*χ* ^2^	0.036	0.009	0.020	0.009	0.018	0.011

## Data Availability

All data are included in the manuscript and/or Supplemental Materials. Original data will be made available upon requests after publication.

## Supplementary Materials

The following supporting information can be downloaded at https://www.mdpi.com/article/10.3390/biom16010137/s1, Figure S1: (a) Calibration curve of doxorubicin (DOX) in PBS buffer (pH 9.5) obtained by UV–VIS spectroscopy at 498 nm, and (b) UV–VIS spectra of all DOX solutions used for the calibration; Figure S2: Calibration curves of doxorubicin (DOX) in PBS buffer at (a) pH 4.2, (c) pH 5.0 and (e) pH 7.4 obtained by UV–VIS spectroscopy at 498 nm, and UV–VIS spectra of all DOX solutions used for the calibration in (b), (d) and (f); Figure S3: SEM and BF-STEM images of (a,b) Fe_3_O_4_, (c,d) MNP-HTCC1 and (e,f) MNP-HTCC2. Images on the left correspond to SEM mode, and those on the right to BF-STEM mode; Figure S4: XRD diffractograms of Fe_3_O_4_, MNP-HTCC1 and MNP-HTCC, Figure S5: Speciation of DOX.
