# Supplementary material for: Magnetic Nanocarriers with ICPTES- and GPTMS-Functionalized Quaternary Chitosan for pH-Responsive Doxorubicin Release
Source: Biomolecules. 2026 Jan 13;16(1):137. doi: 10.3390/biom16010137 (PMC12839120; doi:10.3390/biom16010137)
Supplement: Supplementary file 1 [file biomolecules-16-00137-s001.zip › biomolecules-4017146-supplementary.pdf]

Supplementary Information

Magnetic Nanocarriers with ICPTES- and GPTMS-Functionalized Quaternary Chitosan for pH Responsive Doxorubicin Release

Sofia F. Soares[1]*, Ana L. M. Machado[1,‡], Beatriz S. Cardoso[1, ‡], Diogo Marinheiro[1,2], Nelson Andrade[2,3], Fátima Martel[2,4], Ana L. Daniel-da-Silva [1]*

[1]CICECO-Aveiro Institute of Materials, Department of Chemistry, University of Aveiro, 3810-193 Aveiro, Portugal

[2]Unit of Biochemistry, Department of Biomedicine, Faculty of Medicine of Porto, University of Porto, 4200-319 Porto, Portugal

[3]REQUIMTE/LAQV, Department of Chemical Sciences, Faculty of Pharmacy, University of Porto, 4050-313 Porto, Portugal

[4]Instituto de Investigação e Inovação em Saúde (i3S), University of Porto, 4200-465 Porto, Portugal

* To whom correspondence should be addressed.

E-mail: sofiafsoares@ua.pt; ana.luisa@ua.pt

‡ These authors contributed equally to this work.

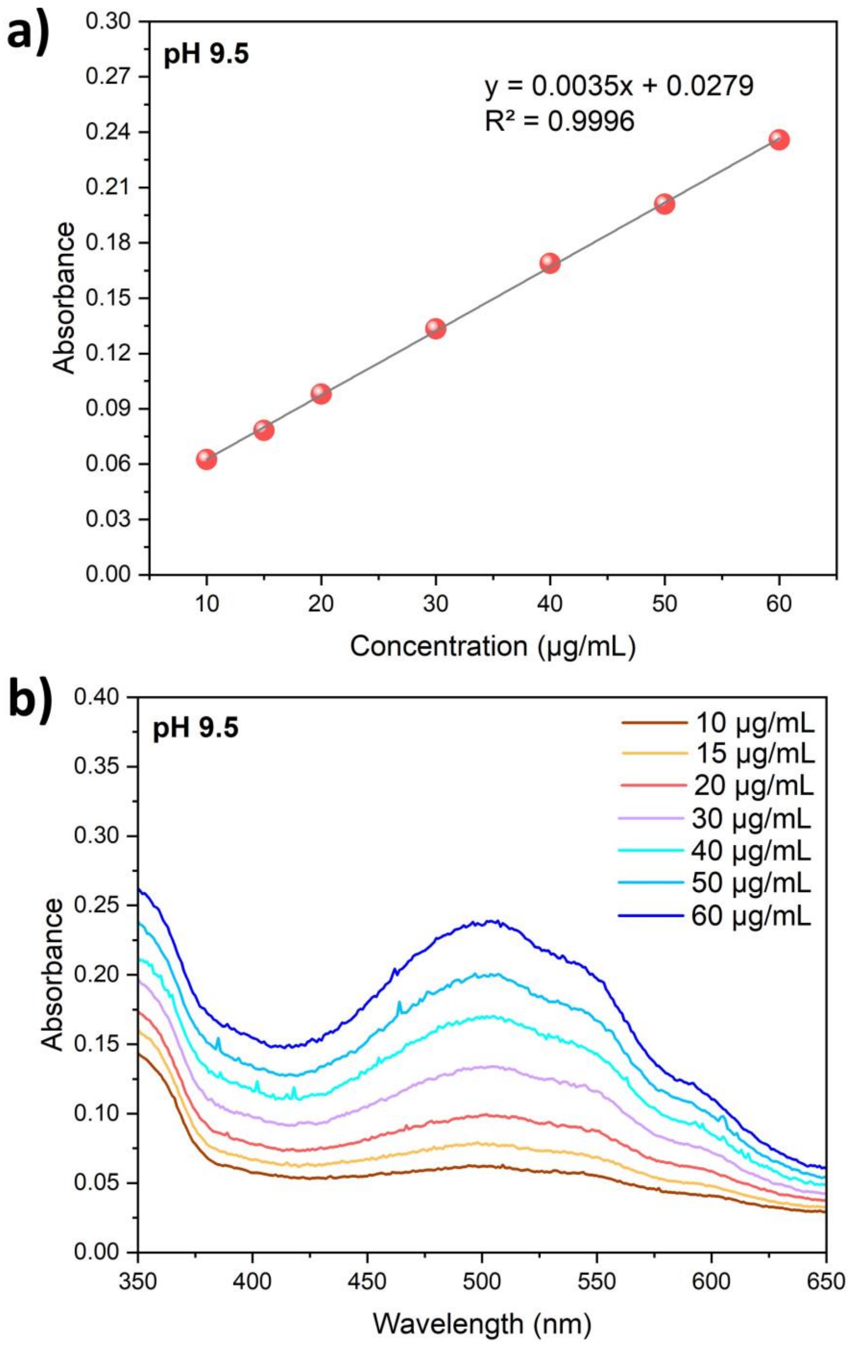

Figure S1. a) Calibration curve of doxorubicin (DOX) in PBS buffer (pH 9.5) obtained by UV–VIS spectroscopy at 498 nm, and b) UV–VIS spectra of all DOX solutions used for the calibration.

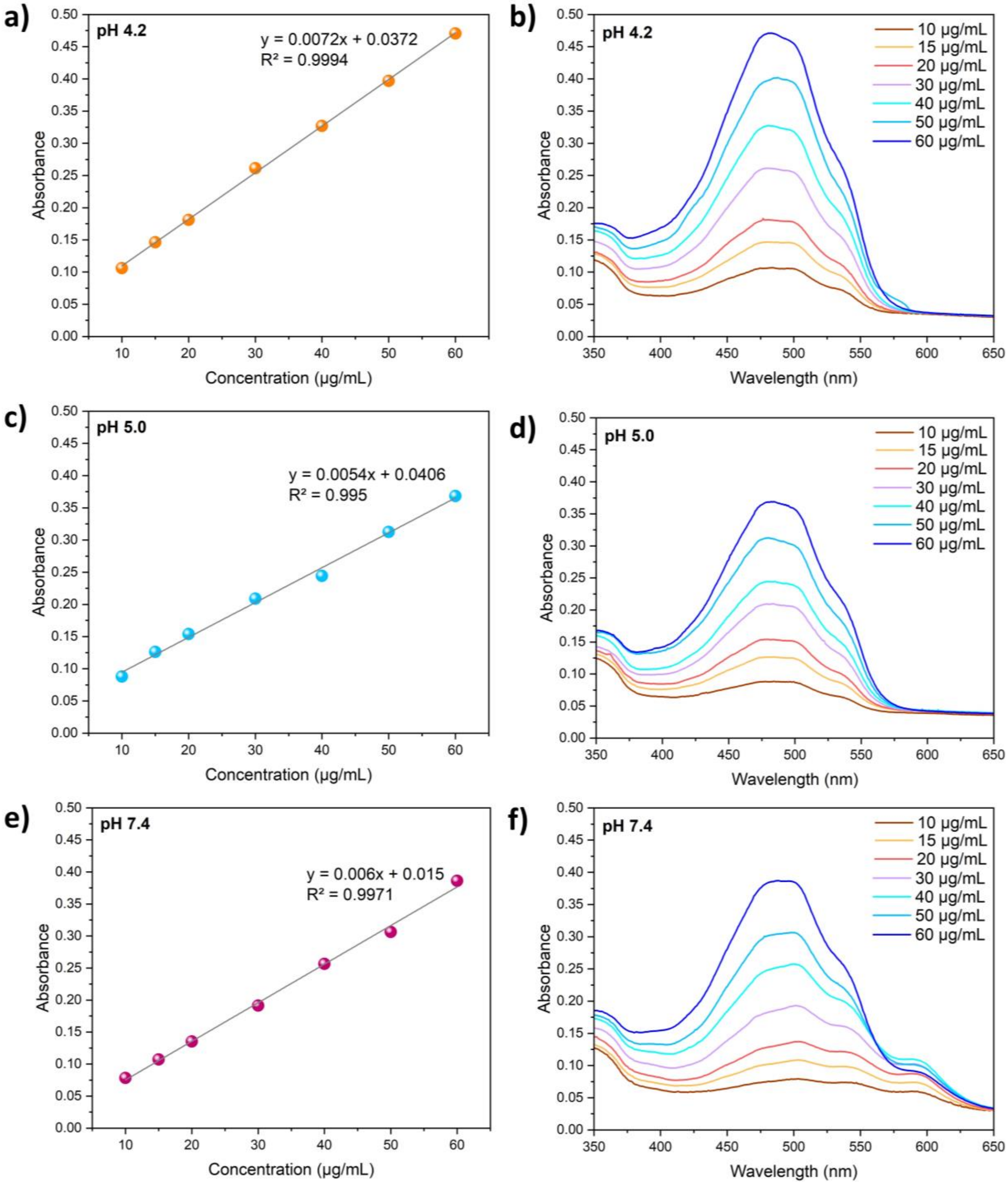

Figure S2. Calibration curves of doxorubicin (DOX) in PBS buffer at a) pH 4.2, c) pH 5.0 and e) pH 7.4 obtained by UV–VIS spectroscopy at 498 nm, and UV–VIS spectra of all DOX solutions used for the calibration in b), d) and f).

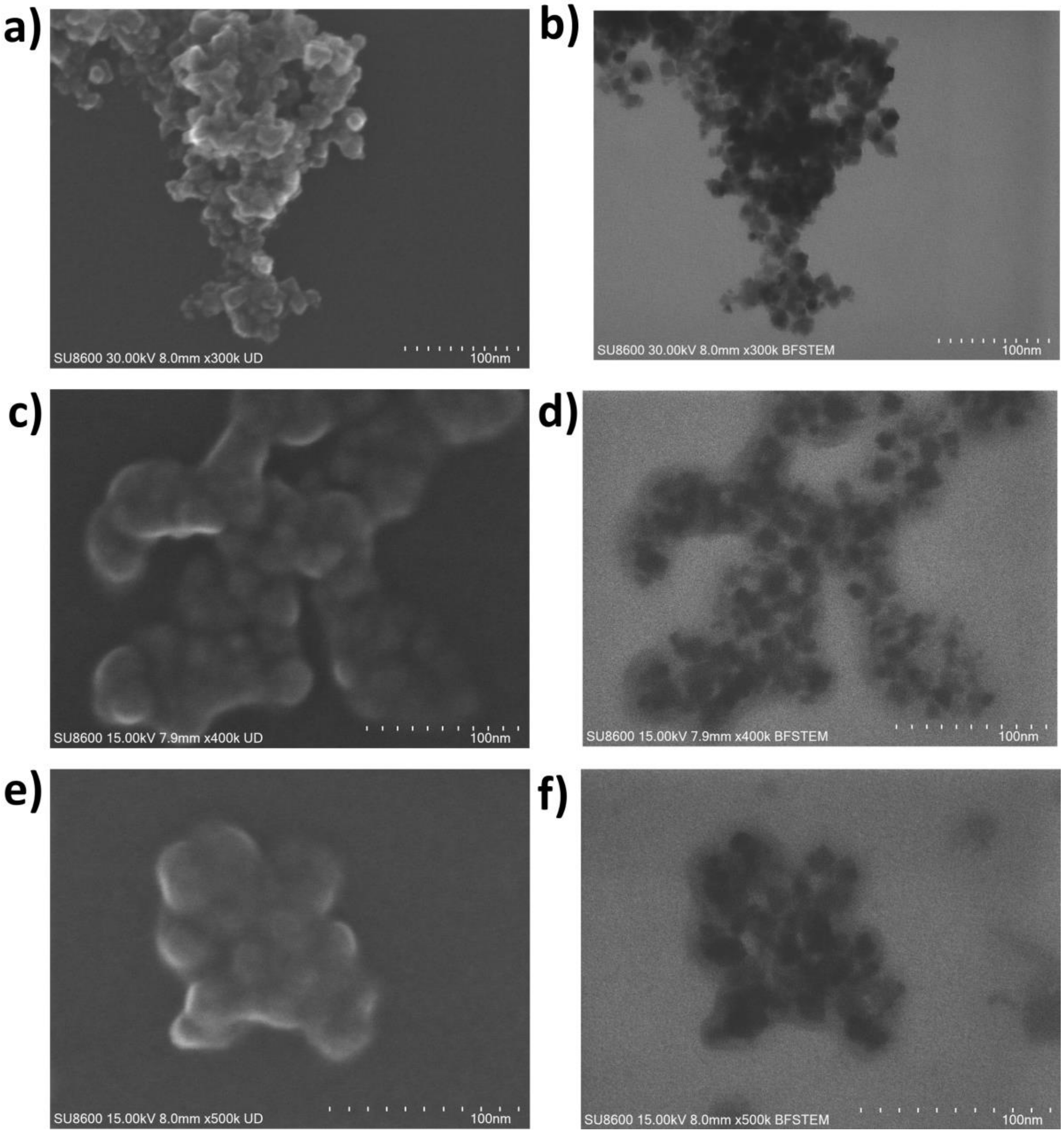

Figure S3. SEM and BF-STEM images of (a,b) Fe_3O_4, (c,d) MNP-HTCC1 and (e,f) MNP-HTCC2. Images on the left correspond to SEM mode, and those on the right to BF-STEM mode.

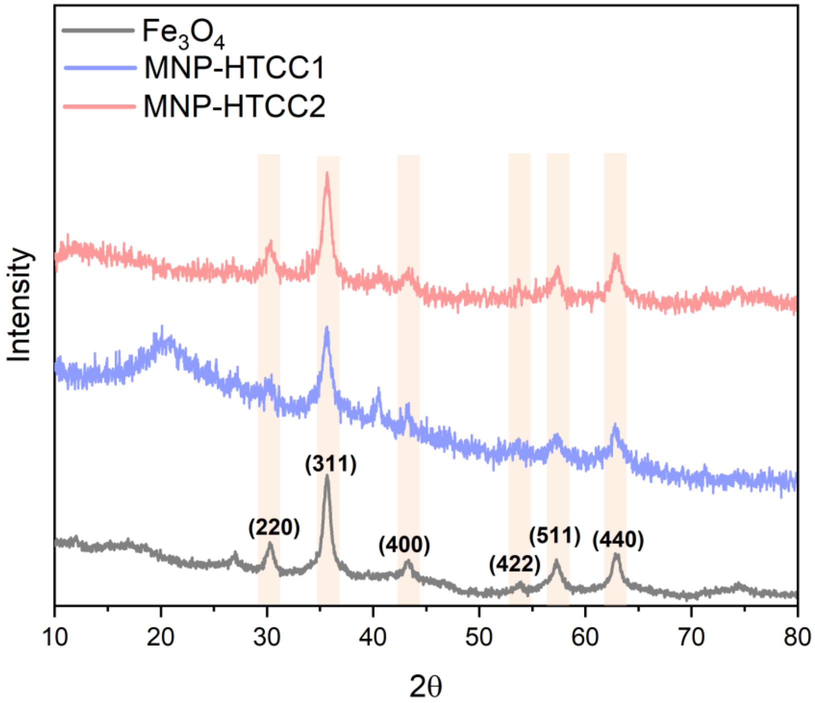

Figure S4. XRD diffractograms of Fe_3O_4, MNP-HTCC1 and MNP-HTCC2.

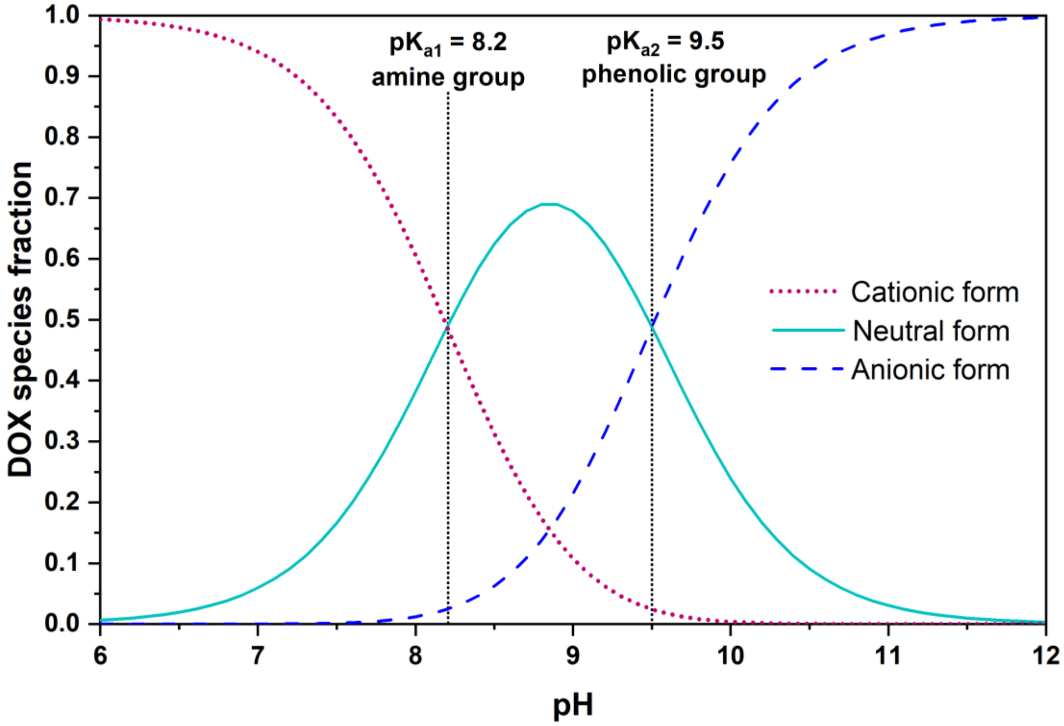

Figure S5. Speciation of DOX.
